# Personalized Nutrition: Tailoring Dietary Recommendations through Genetic Insights

**DOI:** 10.3390/nu16162673

**Published:** 2024-08-13

**Authors:** Saiful Singar, Ravinder Nagpal, Bahram H. Arjmandi, Neda S. Akhavan

**Affiliations:** 1 Department of Health, Nutrition, and Food Sciences, College of Education, Health, and Human Sciences, Florida State University, Tallahassee, FL 32306, USA; ssingar@fsu.edu (S.S.); rnagpal@fsu.edu (R.N.); barjmandi@fsu.edu (B.H.A.); 2 Department of Kinesiology and Nutrition Sciences, School of Integrated Health Sciences, University of Nevada, Las Vegas, NV 89154, USA

**Keywords:** personalized nutrition, nutrigenomics, genetic variability, dietary interventions, chronic disease management, bioinformatics in nutrition

## Abstract

Personalized nutrition (PN) represents a transformative approach in dietary science, where individual genetic profiles guide tailored dietary recommendations, thereby optimizing health outcomes and managing chronic diseases more effectively. This review synthesizes key aspects of PN, emphasizing the genetic basis of dietary responses, contemporary research, and practical applications. We explore how individual genetic differences influence dietary metabolisms, thus underscoring the importance of nutrigenomics in developing personalized dietary guidelines. Current research in PN highlights significant gene–diet interactions that affect various conditions, including obesity and diabetes, suggesting that dietary interventions could be more precise and beneficial if they are customized to genetic profiles. Moreover, we discuss practical implementations of PN, including technological advancements in genetic testing that enable real-time dietary customization. Looking forward, this review identifies the robust integration of bioinformatics and genomics as critical for advancing PN. We advocate for multidisciplinary research to overcome current challenges, such as data privacy and ethical concerns associated with genetic testing. The future of PN lies in broader adoption across health and wellness sectors, promising significant advancements in public health and personalized medicine.

## 1. Introduction

Personalized nutrition (PN) is a discipline that utilizes the unique characteristics of individuals to formulate nutritional approaches aimed at preventing, managing, and treating diseases, as well as enhancing overall health. According to the American Nutrition Association, this field is characterized by three interconnected components: the science and data behind PN, professional education and training in PN, and the application of PN in guidance and therapeutic practices [1]. PN encompasses the use of genetic, phenotypic, biochemical, and nutritional data to analyze their effects on an individual’s health. It also prepares healthcare providers to implement PN strategies in diverse environments and customize interventions to meet specific personal requirements [1]. The International Society of Nutrigenetics/Nutrigenomics (ISNN) offers insights into PN, highlighting how an individual’s genetic makeup and a range of biological and cultural differences, such as food intolerances, preferences, and allergies, influence their response to nutrients [2]. PN operates on the principle that individual genetic variations can influence how certain foods or amounts of nutrients modify one’s risk of disease [2]. The breadth of PN is enhanced by incorporating a variety of phenotypic data, including measurements of body composition, levels of physical activity, clinical indicators, and biochemical markers that assess nutritional status, along with genomic information, to deliver more customized dietary recommendations [3]. This approach is seen to exist at multiple levels, from internet-delivered services to the use of genomic data in crafting personalized dietary advice [3].

The historical perspective on dietary recommendations reveals a shift in focus from preventing nutrient deficiencies to addressing chronic diseases associated with dietary excesses. Initially, dietary guidelines were developed to ensure nutritional adequacy and prevent deficiencies, particularly in the context of food scarcity. As societies transitioned from scarcity to abundance, the prevalence of chronic diseases such as heart disease, obesity, and diabetes increased, prompting a change in dietary guidance toward the prevention of these conditions [4,5,6]. Early dietary recommendations emphasized the consumption of animal products and were less concerned with chronic disease prevention. However, as evidence accumulated linking diet to chronic disease risk, there was a growing consensus on the benefits of plant-based diets, including vegetarian, Mediterranean, and Asian diets, which were associated with lower rates of chronic diseases [7]. The limitations of historical dietary recommendations include a reliance on evidence that may not have been robust or comprehensive, leading to guidelines that may not have been fully supported by the available science (Table 1). For example, the initial Dietary Goals for Americans proposed changes in macronutrient consumption without sufficient evidence to conclusively recommend such shifts [5]. Additionally, the application of these guidelines to diverse populations, including children, was often based on inferences rather than direct evidence of benefit [4]. Furthermore, the methods used to assess dietary intake, such as Food Frequency Questionnaires, have been criticized for their inaccuracy and the potential for recall bias, which can undermine the validity of diet-disease relationships established in epidemiological studies [8].

The promise of genomics in enhancing dietary interventions lies in the potential to tailor nutrition based on individual genetic variability. Nutrigenomics explores the interaction between food and our genetic makeup, examining how our individual genetic variations influence our response to nutrients in our diet. This field holds promise for tailoring dietary guidelines to individual health needs, potentially enhancing health outcomes. For instance, genome-wide single nucleotide polymorphism (SNP) data can be used to create personalized dietary recommendations, taking into account an individual’s genetic variability at multiple SNPs [9]. Incorporating genomics into the field of nutritional sciences can enhance the efficacy of nutritional interventions. This approach allows for a deeper understanding of the intricate relationships between dietary components and the human genome across various states of health and illness [10]. This approach could pave the way for creating dietary guidelines that are highly predictive, reducing the likelihood of unforeseen outcomes and considering the impact of human genetic differences [10]. Moreover, the inclusion of genomic information in dietary interventions has been shown to enhance the accuracy of weight loss models, indicating the efficacy of coaching informed by participants’ genomic risk [11]. This suggests that genomics can play a role in adopting changes beyond population-wide healthy eating guidelines, potentially mitigating the prevalence of obesity and other chronic diseases [12]. However, it is important to note that while the field of nutritional genomics holds great promise, it is still emerging, and more research is needed to fully realize its potential in clinical practice. The ISNN recognizes the ethical, practical, and scientific obstacles that need to be overcome to successfully apply the findings from gene–nutrient interaction research into reliable practice guidelines for PN [12,13].

## 2. Genetic Variability and Nutrient Metabolism

**Figure 1:**
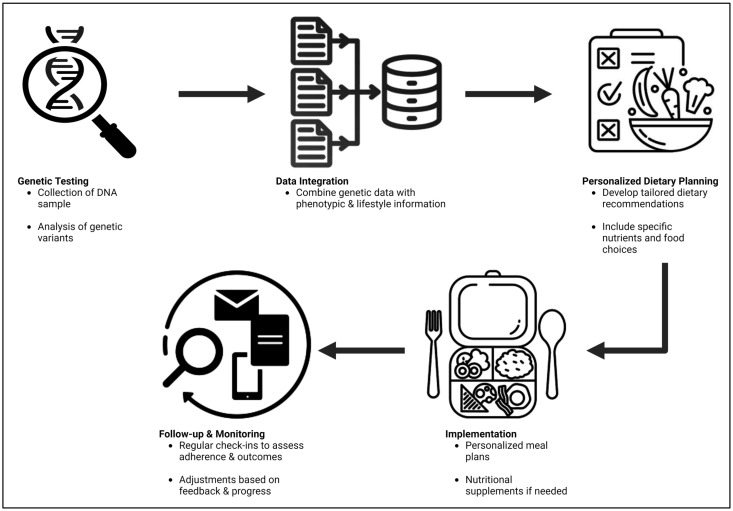
Workflow of a personalized nutrition program.

Human genetic variation encompasses the differences in DNA sequences among individuals, which contribute to the diverse phenotypic characteristics observed in the human population. Every human genome has more than 3 million single nucleotide variants (SNVs) compared to the reference genome, and roughly 1% of a person’s genome varies from this reference sequence [14]. These types of genetic variations encompass SNPs, insertions and deletions (indels), copy number variations (CNVs), and structural changes like inversions and complex rearrangements [14,15,16]. The average human gene contains numerous biallelic polymorphisms, with a subset found in coding regions that can affect protein function [17]. The distribution of these genetic variants varies among populations, with some being common and others rare. Rare variants often show substantial geographic differentiation, influenced by factors such as evolutionary conservation, coding consequence, and purifying selection [15]. The 1000 Genomes Project has created a detailed map of human genetic diversity, encompassing SNPs, short insertions and deletions, and larger structural deletions. It covers as much as 98% of SNPs that are accessible and have a frequency of at least 1% within populations that are closely related [15]. This variation reflects the history of human migration, population dynamics, and adaptation to different environments [18,19]. Understanding human genetic variation is crucial for the study of genetic diseases, the development of personalized medicine, and the implementation of genomic-informed dietary interventions (Figure 1). It enables the detection of genetic factors that influence susceptibility to diseases and reactions to treatments, including dietary interventions.

Genetic factors can significantly influence nutrient metabolism, and several examples are highlighted in the literature. Variants in the methylenetetrahydrofolate reductase (MTHFR) gene can affect folate metabolism and are linked to cardiovascular disease and diabetes. Individuals with certain SNPs in this gene may have altered responses to folate intake and could benefit from tailored folate supplementation [20,21,22]. Selenium metabolism can also be influenced by genetic variation, with SNPs affecting the response of selenoprotein expression or activity to selenium supplementation [20]. Genetic variations in the beta-carotene oxygenase 1 (BCMO1) gene, which is critical for beta-carotene metabolism, can lead to variability in plasma carotenoid levels and may have clinical significance, such as the development of liver steatosis independent of dietary vitamin A [23,24]. Furthermore, genetic variations in genes that play a role in lipid metabolism, including cholesteryl ester transfer protein, lipoprotein lipase, low-density lipoprotein receptor, and apolipoprotein E, have the potential to influence the risk of coronary artery disease [25,26,27]. Personalized dietary recommendations that consider these genetic differences may prove beneficial [12]. Additionally, SNPs in genes related to choline and folate metabolism can determine the dietary requirement for choline, which is crucial for liver function and fetal development [28]. These examples underscore the importance of considering genetic factors when evaluating nutrient metabolism and the potential for PN interventions to optimize health outcomes based on individual genetic profiles.

Case studies in the literature illustrate the impact of genetics on dietary needs through the lens of nutrigenetics. For example, individuals with the homozygous mutation (TT) in the MTHFR gene (MTHFRAla222Val, C > T polymorphism) have increased requirements for folate due to altered folate metabolism. These individuals might require a greater intake of folate than the suggested dietary allowances (RDAs) to reduce their susceptibility to diseases associated with folate deficiency [12]. Similarly, individuals with Down syndrome, who possess an additional copy of chromosome 21, demonstrate higher needs for zinc and folate because of the heightened expression of cystathionine-β-synthase, a key enzyme in folate metabolism [12]. Another case study examines the APOA1 polymorphism (G > A). It reveals that individuals with the A allele experience elevated levels of HDL cholesterol following increased intake of long-chain omega-3 polyunsaturated fatty acids (PUFAs), while those with the GG genotype do not demonstrate this advantage [12]. Additionally, people carrying significant risk alleles for serum- and glucocorticoid-inducible kinase 1 (SGK1) might show increased systolic blood pressure when they consume a diet high in salt [12]. These examples underscore the importance of considering genetic variations when assessing dietary needs and the potential for PN to optimize health outcomes based on individual genetic profiles (Table 2).

## 3. Current Research in Nutrigenomics

Key studies have identified gene–diet interactions that influence the risk of developing conditions such as obesity and type 2 diabetes mellitus (T2DM). For instance, dietary patterns that emphasize whole grains, vegetables, and fruits, and limit total and saturated fats, such as the Mediterranean and DASH diets, have demonstrated the potential to lower the risk of obesity for people with high genetic predisposition scores for obesity, especially in those with risk alleles of FTO rs9939609, rs1121980, and rs1421085 [29]. However, findings related to other SNPs in genes like MC4R, APOA5, and PPARG were inconclusive [29]. In the context of T2DM, gene–macronutrient interactions have been studied, with several genetic variants in or near genes like TCF7L2, GIPR, CAV2, and PEPD showing potential interactions with macronutrients such as carbohydrates, fats, saturated fats, dietary fiber, and glycemic load. However, the large-scale EPIC-InterAct study did not replicate these interactions [30]. Research on gene–diet interactions has extended into the realm of maternal–child health, examining issues such as gestational diabetes, pregnancy-induced hypertension, recurrent miscarriages, iron deficiency anemia, and excessive weight gain during pregnancy. Findings from these studies indicate that insights into gene–diet interactions might contribute to tailored nutritional strategies for mothers and their children [33]. Overall, while there is evidence of gene–diet interactions affecting metabolic health, the findings are often specific to certain populations or genetic variants and may not be universally applicable (Table 3). Further research is needed to better understand these interactions and to develop PN strategies based on genetic profiles [29,30,33].

Technological advancements in genetic testing and bioinformatics have significantly enhanced the ability to identify genetic biomarkers that inform on disease susceptibility, progression, and response to therapy. The development of molecular point-of-care tests (POCTs) has been particularly notable, with microfluidic technologies and novel amplification methods enabling rapid genetic testing at the point of care, which is expected to further the adoption of personalized medicine methods [34]. Advances in next-generation sequencing (NGS) technologies, such as massively parallel sequencing, have significantly decreased the costs and accelerated the process of DNA sequencing. This advancement enables both whole-genome sequencing (WGS) and the targeted sequencing of particular genomic areas. As a result, NGS has begun to integrate into clinical settings, offering improvements in the diagnosis, prognosis, and treatment selection for numerous diseases [35,36,37]. Artificial intelligence (AI) is being integrated into clinical laboratory genomics to manage the “big data” generated by these technologies. AI enhances genetic research by helping identify variants in DNA sequences, predicting how these variants could affect protein structure and function, and linking genomic data with clinical insights. This support bolsters the ability of geneticists to convert intricate data into practical information for managing patient care [38]. Advancements in long-read sequencing and long-range mapping technologies are enhancing genomic diagnostics by enabling the identification of a broader range of variants and providing a more holistic perspective of transcriptomes and epigenomes. To fully capitalize on these technologies’ distinct features and tackle their complex error profiles, new bioinformatics strategies are essential [39].

In nutritional research, several genetic markers have been identified as significant in understanding gene–diet interactions. Variants of the fat mass and obesity-associated (FTO) gene, including rs9939609, rs1121980, and rs1421085, have been linked to an increased risk of obesity. These genetic markers have shown interactions with dietary patterns that are high in whole grains, vegetables, and fruits, and low in both total and saturated fats [29]. Other genes commonly studied include MC4R, PPARG, and APOA5, although findings related to their interaction with diet on overnutrition have been inconclusive [29]. The gene responsible for fibroblast growth factor 21 (FGF21) has been correlated with macronutrient intake. Specifically, a variation located at the chromosome 19 locus (rs838145) has been associated with increased carbohydrate intake and decreased fat consumption [40]. Additionally, the FTO variant (rs1421085) is associated with higher protein intake, independent of body mass index [40]. The ISNN emphasizes the significance of providing dietary recommendations customized to genetic variations, including those found in genes crucial for lipid metabolism (such as cholesteryl ester transfer protein, lipoprotein lipase, low-density lipoprotein receptor, and apolipoprotein E), which could impact the susceptibility to coronary artery disease [2]. Furthermore, specific polymorphisms have been shown to interact with nutrient intake, influencing obesity and abdominal obesity. For instance, the presence of the minor allele (A) of the Ca binding protein 39 (CAB39) rs6722579 gene variant is linked to an increased susceptibility to abdominal obesity in those who exceed the Dietary Reference Intakes (DRIs) for fat consumption [41]. Conversely, individuals carrying the minor allele (T) of the carboxypeptidase Q (CPQ) rs59465035 gene tend to exhibit reduced susceptibility to abdominal obesity, particularly in cases of higher vitamin C consumption [41]. These genetic markers are instrumental in advancing the field of PN, where dietary recommendations can be tailored to an individual’s genetic makeup to optimize health outcomes.

## 4. Methods in Personalized Nutrition Research

The methodologies used in studying nutrigenomics involve a range of high-throughput omics technologies. Transcriptomics, proteomics, and metabolomics are key approaches employed to assess the responses of biological systems to dietary interventions and to understand the interactions between diet and genes [42,43,44,45]. Transcriptomics analyzes genome-wide gene expression changes and has been the most frequently applied technique in nutrigenomics research [45]. Proteomics explores the comprehensive array of proteins produced by a genome, cell, tissue, or organism, a profile that can be influenced by nutritional intake [43]. Metabolomics delves into the distinct chemical imprints left by cellular activities, focusing on the analysis of their profiles of small-molecule metabolites [43,44]. Microarray technology is another powerful tool used in nutrigenomics to evaluate gene expression profiles globally and to understand the regulation of gene transcription by nutrients or dietary bioactive compounds [46]. Additionally, advances in bioinformatics are crucial for the integration and interpretation of the vast amounts of data generated by these omics technologies, allowing for the investigation of complex gene–nutrient interactions and the development of PN strategies [43,47]. These methodologies collectively contribute to the understanding of how dietary components can influence gene regulation, protein expression, and metabolite production, which are central to the field of nutrigenomics and the pursuit of PN.

Designing studies to evaluate genetic and dietary interactions presents several challenges. One major issue is the complexity of gene–nutrient interactions, which increases the dimensionality of the problem, making it difficult to approach these interactions at the population level [48]. Additionally, accurately assessing dietary intake in population studies is complex due to the limitations of current dietary assessment tools like food frequency questionnaires, 24 h food recalls, and diet records. These tools may not be reliable or sensitive enough to capture long-term intake accurately, which is necessary to establish gene–nutrient associations [49]. Another challenge is the small effect size of common genetic variations and the complexity of establishing associations between lifestyle factors and the likelihood of developing obesity in the future. This requires an analytical method that relies on clearly defined prior probabilities to minimize the risk of erroneous findings [50]. Moreover, gene–environment interaction studies must address design and analytical challenges such as confounding and selection bias, measurement accuracy of exposures and genotypes, and the assumptions surrounding biological factors [51]. Furthermore, the genetic architecture of nutrition-related diseases is complex, involving multiple genes and interactions that cannot be explained by single polymorphisms. This complexity requires the use of whole-genome analysis to understand gene interactions and pathways influencing nutritional metabolism [12]. Finally, the use of Mendelian randomization can help to strengthen causality in nutrition research, but it requires careful selection of genetic markers to avoid biases present in observational studies [52].

Analyzing complex genetic data to evaluate gene–diet interactions involves employing a variety of statistical approaches that address the high dimensionality and complexity of the data. Various methodologies such as multifactor dimensionality reduction, grammatical evolution neural networks, random forests, focused interaction testing framework, step-wise logistic regression, and explicit logistic regression are employed for identifying main effects and gene–gene interactions [53]. These methods have their strengths and weaknesses, and their relative success is context-dependent. Methods that leverage summary association data, such as those developed to analyze genome-wide association studies (GWAS) data, are also employed. These methods are particularly useful when individual-level genetic data are not accessible due to privacy concerns or other logistical considerations [54]. In research that deals with pedigrees or data that is structured by population, statistics known as “burden” and kernel statistics have been formulated. These statistics are designed to adjust for the effects of shared genetic heritage and the linkage disequilibrium present among genetic markers [55]. These techniques enhance traditional approaches for unrelated case-control data by incorporating known familial connections. Family-based association studies that utilize next-generation sequencing data can leverage various kinds of family and unrelated individual data collected from diverse population structures [56]. This method is beneficial for conditions influenced by both common and rare genetic variations. It is also essential to employ statistical techniques to analyze complex correlation patterns in extensive pharmacogenomic datasets. These techniques encompass the estimation of large covariance matrices, conducting broad-scale simultaneous tests to identify genes that show significant differential expression, and selecting variables in high-dimensional spaces [57]. The statistical approaches for analyzing complex genetic data in nutrigenomics research are diverse and must be carefully chosen based on the specific context of the study, including the genetic architecture of the trait of interest, the type of dietary exposure, and the population structure.

## 5. Practical Applications of Personalized Nutrition

PN is being implemented in clinical settings through the development and use of tools that integrate individual genetic, phenotypic, and lifestyle data to tailor dietary advice. Clinical Nutritional Information Systems (CNIS) have been implemented to assist hospital dietitians in providing PN assessments, particularly for inpatients requiring cancer nutrition counseling [58]. These systems enhance the standardization of nutritional intervention and monitoring, improve the quality of nutritional interventions through precise calculations and patient information verification, and decrease the time needed for tasks like manual documentation [58]. Additionally, the Academy of Nutrition and Dietetics, along with the American Council on Exercise, have established evidence-based practice guidelines that advocate for tailored nutrition and physical activity interventions. These guidelines underscore the significance of customizing interventions to fit the specific needs of individuals, taking into account comprehensive client data such as genetic information, to guide care [59]. Furthermore, nutrigenomics technology interface tools are being compared and developed to facilitate the translation of nutrition-related gene test information into actionable dietary plans. These tools aim to support health professionals in delivering PN interventions that are more effective in improving health outcomes compared to standard approaches [60]. In critical care settings, PN therapy recommendations incorporate recent literature and guidelines from American/European societies, suggesting the use of indirect calorimetry to measure energy expenditure and guide the personalization of nutrition therapy, including the timing and dosing of macronutrients [61].

Commercially available genetic testing for nutritional advice has expanded with the advent of direct-to-consumer (DTC) nutrigenetics testing services. These services offer nutritional recommendations tailored to a person’s genetic makeup, determining dietary plans from a limited set of genetic markers [62]. Companies such as 23andMe have received FDA authorization to market DTC genetic tests, including reports on carrier status, wellness, traits, and ancestry, although they are clear that their tests are not intended to diagnose health conditions or provide dietary advice [12]. Although debates persist about the effectiveness of individual genetic variants compared to genetic risk scores, and whether DNA-based dietary guidance can truly inspire beneficial behavioral changes, there is supportive evidence that genetic testing can inform dietary suggestions to improve health and performance [63]. However, it is important to note that the scientific evidence supporting gene–diet interactions and the effectiveness of gene-based dietary recommendations is still evolving, and more research is needed to establish clinical evidence for these PN approaches [31]. Physicians should be aware that while these tests can provide insights into genetic predispositions, they should be interpreted with caution and in the context of a comprehensive approach to patient care that considers the totality of genetic, phenotypic, and environmental factors influencing health.

**Figure 2:**
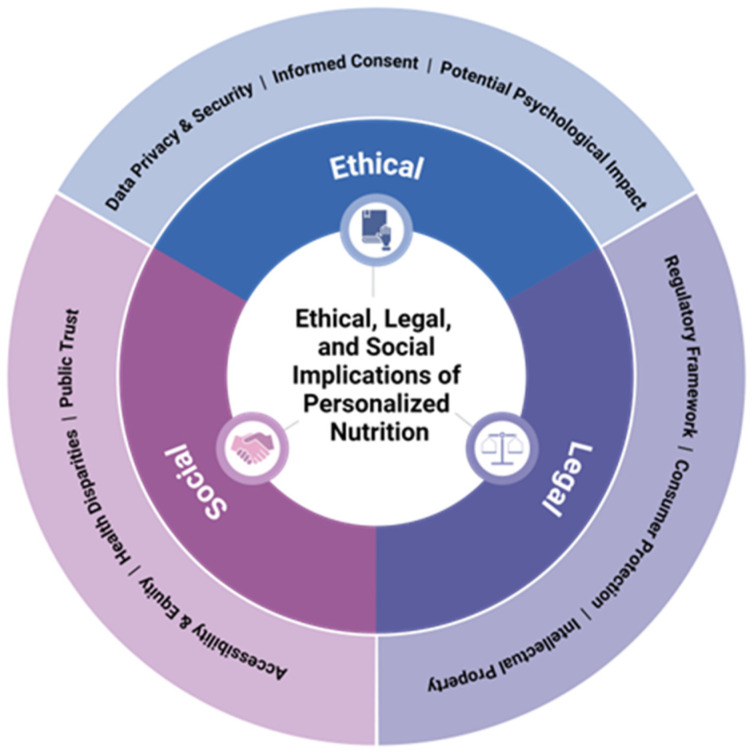
Ethical, legal, and social implications of personalized nutrition.

The ethical, legal, and social implications of PN are multifaceted (Figure 2). Ethically, the use of genetic profiles in nutritional advice raises concerns about privacy, informed consent, and the potential psychological impacts of genetic information. Legally, there is a lack of specific regulations for genetic testing in the context of PN, which can lead to consumer protection issues, particularly with direct-to-consumer (DTC) genetic tests. Socially, there is the risk of exacerbating health inequalities if PN services are not accessible to all segments of the population. The ISNN has discussed these issues, noting the importance of integrating nutrigenetic approaches with cultural, emotional, and ethical aspects of food, and the need for individually tailored nutritional advice to extend beyond single nutrient recommendations [12]. Furthermore, there is a need for healthcare professionals, including registered dietitian nutritionists (RDNs), to be involved in the delivery of PN to ensure that ethical and legal standards are met. This involvement includes protecting individual privacy, transparently communicating effects, and identifying potential beneficiaries of PN technologies [32]. The ISNN also emphasizes the importance of considering the social aspects of eating when providing PN advice [12]. PN must be approached with careful consideration of ethical, legal, and social implications, ensuring that services are delivered responsibly and equitably, with appropriate involvement of healthcare professionals to guide and regulate practice [12,32].

## 6. Impact on Disease Prevention and Management

PN aims to combat and control chronic conditions like cardiovascular diseases and diabetes by customizing dietary strategies based on a person’s genetic makeup, metabolic characteristics, and environmental factors. The convergence of genomics, metabolomics, and gut microbiome advancements has enabled the application of precision nutrition strategies to effectively prevent and manage type 2 diabetes [64]. Research in nutrigenomics has uncovered specific genetic variants that affect the intake and metabolism of nutrients, indicating that individual responses to dietary interventions can vary based on genetic variability [64]. In the context of cardiovascular disease prevention, the PREDIMED study has demonstrated the clinical relevance of gene–diet interactions, suggesting that PN could be a more effective tool for chronic disease prevention than traditional one-size-fits-all recommendations [65]. Additionally, the concept of metabotyping involves customizing dietary recommendations based on the metabolic characteristics of different groups, which could be especially significant in preventing cardiometabolic diseases [66]. The ISNN supports the notion that PN, which includes the consideration of genotype, can be more effective in improving public health and may be beneficial in long-term weight control [12,67]. However, the use of nutrigenomics and nutrigenetics in managing specific diseases such as cardiovascular disease remains in a nascent phase. An integrated strategy that incorporates lifestyle changes and tailored dietary guidance, reflecting an individual’s phenotype and genotype, is essential [12]. PN, informed by genetic and omic data, offers a promising approach to the prevention and management of chronic diseases by providing more precise dietary recommendations that consider individual variability [12,64,65,66,67,68].

Nutrigenomics plays a pivotal role in weight management and obesity prevention by elucidating the interplay between diet and an individual’s genetic makeup. The field has identified specific genetic markers that influence an individual’s response to dietary components, which can be leveraged to tailor weight management strategies. For instance, variations in the FTO gene have been associated with differential responses to macronutrient intake and susceptibility to obesity [69,70]. Nutrigenomic analysis has also demonstrated that certain dietary supplements, such as hydroxycitric acid and niacin-bound chromium, can alter gene expression related to adipogenic and lipolytic pathways, potentially offering new avenues for obesity management [71]. Furthermore, the integration of nutrigenetic and nutrigenomic approaches can enhance the precision of obesity care by considering both genetic predispositions and gene expression changes in response to diet [70]. This includes the assessment of polymorphisms that influence energy homeostasis and body composition, as well as the analysis of epigenetic mechanisms such as DNA methylation and microRNA expression profiles [72]. Clinical trials, such as the Nutrigenomics, Overweight/Obesity and Weight Management (NOW) trial, have provided evidence that nutrigenomics interventions can lead to greater reductions in body fat percentage compared to standard interventions [73]. This supports the potential of nutrigenomics to optimize weight management strategies. Nutrigenomics offers a promising approach to weight management and obesity prevention by providing insights into how genetic and epigenetic factors interact with dietary intake, thereby enabling the development of personalized dietary strategies that can improve obesity-related outcomes.

In the context of PN for weight management and obesity prevention, a study by Arkadianos et al. showed that integrating genetic data to tailor dietary plans enhances long-term weight management and prevents obesity [74]. Patients who underwent a nutrigenetic test screening for 24 variants across 19 metabolism-related genes and received tailored dietary guidance demonstrated enhanced adherence, more substantial long-term reductions in BMI, and better blood glucose outcomes compared to those without genetic testing. Another study by Ramos-Lopez et al. developed a model that integrates genetic, phenotypic, and environmental data to customize low-calorie diets with varying macronutrient compositions [75]. The study found that various genetic, phenotypic, and external factors influence the reduction in BMI based on whether a moderately high-protein hypocaloric diet or a low-fat diet is followed. Using a comprehensive approach could enhance the customization of dietary recommendations for controlling obesity through the use of precision nutrition variables. Additionally, the Food4Me study investigated the relationships and possible interactions between adherence to the Mediterranean Diet and genetic predispositions during an online nutritional intervention [76]. The study revealed that greater adherence to the Mediterranean Diet leads to positive effects on metabolic markers, which may be influenced by genetic factors in certain specific indicators. These studies illustrate the successful application of nutrigenetic testing and PN interventions in clinical settings, leading to improved outcomes in weight management and obesity prevention.

## 7. Challenges and Controversies

The scientific and technological limitations include the complexity of gene–nutrient interactions and the challenges in accurately assessing long-term dietary intake. Many diseases linked to nutrition are intricate and involve multiple genes, indicating that single polymorphisms are insufficient to comprehensively elucidate the associated conditions and traits. Whole-genome analysis could aid in grasping how genes interact and how pathways affect nutritional metabolism. However, continual enhancements in genetic testing and analysis are essential to underpin the forthcoming advancements in nutrigenomics [12]. Additionally, the tools for assessing food intake, such as 24 h food recalls, diet recalls, and food frequency questionnaires, have not developed as rapidly as omics technologies and often lack reliability [12]. Furthermore, there is a need for robust and reproducible results, economical omics technologies, and improvements in research methodology, including rigorous study design, alongside advanced analyses and comprehension of high-dimensional data [64]. The field also faces challenges in defining optimal responses due to a lack of key health biomarkers and signatures [77]. These limitations highlight the need for continued research and development to enhance the efficacy and practical application of PN in clinical and public health settings.

Concerns about privacy and data security are significant, as the integration of genetic data with dietary information increases the sensitivity of the data being handled. The ISNN has highlighted the need for a better understanding of ethical issues and the development of a robust regulatory framework before PN can become commercially viable for everyone [12]. The concerns are centered around the secure handling of health data, particularly with the rise of DTC genetic tests and online service delivery systems. Technological progress is crucial to safeguarding the privacy of online service delivery systems and securing the data collected during the creation of tailored nutrition therapies [78]. Consumers have reservations about the ability of service providers to ensure data security, and there is a demand for an efficacious, transparent, and trustworthy regulatory framework to alleviate these concerns [79]. Additionally, further advances in data interpretation tools are necessary to ensure the effective delivery of information acquired from tests and technologies to consumers [12]. Protecting individual data privacy and acting responsibly are paramount as PN services develop and become more widely available [80].

Skepticism in the medical community and among the public concerning is multifaceted. While there is considerable interest in PN, controversies arise regarding the extent of genetic influence on individual responses to diet, the effectiveness of single genetic markers versus genetic risk scores, the capacity of DNA-informed dietary guidance to promote beneficial behavioral shifts and health outcomes, and the impact of genetic insights on the type of dietary recommendations provided [63]. Despite these controversies, a solid body of evidence shows that genetic testing for PN can guide dietary recommendations to improve health and performance [63]. Public acceptance of PN is influenced by concerns about the secure handling of health data and the ability of service providers to ensure privacy [78]. There is also a general agreement on the promise of nutrigenomics for better understanding diet–health relationships, but less consensus exists on the potential of consumer applications such as PN [81]. Negative consumer opinion and concerns about how genetic information is used and held pose potential barriers to the application of nutrigenomic interventions [82]. The ethical and legal issues around PN are recognized, and there is a need for guidance on how to address these issues and regulate practice [83,84]. The North American division of the International Life Sciences Institute has put forth guiding principles for adopting PN strategies. These underscore the significance of utilizing personalized data based on scientific evidence to encourage dietary changes that could lead to measurable improvements in health [80].

Regulatory issues and standardization of practices are evolving areas with significant implications for clinical implementation. In the United States, the Food and Drug Administration (FDA), the Centers for Medicare & Medicaid Services (CMS), and the Federal Trade Commission (FTC) are involved in regulating genetic tests, which are integral to PN. The FDA has broad authority under the Federal Food, Drug, and Cosmetic Act and regulates tests sold as kits to multiple laboratories. However, laboratory-developed tests (LDTs), which are more common, are not currently regulated by the FDA, although there has been discussion about future regulation [12]. The CMS ensures clinical laboratories comply with the Clinical Laboratory Improvement Amendments (CLIA) of 1988, focusing on the qualifications of technicians and the quality control of lab processes rather than the clinical meaningfulness of genetic tests [12]. In Europe, the regulatory framework is less clear, with no specific legal instruments dealing with PN. Different countries have various regulations regarding genetic tests, with some requiring informed consent and genetic counseling, while others have no specific legislation for direct-to-consumer tests but require medical physician involvement if the test is of a medical nature [12]. These regulatory nuances highlight the need for standardized practices and guidelines to ensure the responsible delivery of PN services, protect consumer privacy, and maintain data security. The ISNN emphasizes the integration of nutrigenetic approaches with cultural, emotional, ethical, and sensual understandings of food, suggesting that PN should extend beyond single nutrient recommendations to include meals and recipes [12].

## 8. Future Perspectives

Emerging trends in research and technology of PN are being driven by advancements in various “omics” technologies and digital health tools (Table 4). The integration of genomics, proteomics, and metabolomics, alongside wearable technologies for tracking dietary intakes and physiological parameters, is advancing the concept of PN [85]. The detailed characterization of food molecules, including their chemical makeup, physical-chemical structure, and biological characteristics, is gaining importance. This process is crucial for aligning human genetic and physical traits with specific foods that promote better physiological health outcomes [86]. The use of AI and machine learning (ML) algorithms is also a growing trend, enabling the integration of large datasets from omic profiles with personal and clinical measures to provide PN advice [87]. Wearable and mobile sensors are being developed to monitor nutritional status at the molecular level, offering non-invasive, real-time dietary information that supports dietary behavior change toward managed nutritional balance [88]. However, there are challenges in implementing these technologies in clinical and public health settings, including the need for robust and reproducible results, cost-effectiveness, and methodological improvements in study design and data interpretation [64]. Despite these challenges, these technologies hold the potential to transform dietary decision-making and improve population health through precision nutrition [89].

The potential for integrating AI and ML in PN is substantial, as these technologies can manage and analyze the large and complex datasets characteristic of nutrigenomics and dietary patterns. AI and ML can improve screening and assessment in clinical nutrition, predict clinical events and outcomes, and integrate diverse data sources such as microbiota and metabolomics profiles with clinical conditions [90]. These technologies introduce innovative approaches in dietary assessment, recognizing food items, predicting models for disease prevention, and diagnosing and monitoring diseases [91]. ML, in particular, has shown promise in developing predictive models suitable for precision nutrition, facilitating the incorporation of complex features and allowing for high-performance PN approaches [92]. Moreover, AI and ML can support the design of personalized dietary plans based on individual genetic data, potentially improving patient outcomes in various health conditions, including weight management and chronic disease prevention [93,94]. However, ethical considerations and the limitations of AI must be considered, including data privacy, security, and the need for transparency in algorithmic decision-making [90]. The incorporation of AI into nutrition offers considerable potential. However, it is crucial to manage these challenges with care to improve individual nutritional results and effectively refine dietary recommendations.

The potential for widespread implementation of PN in the healthcare and wellness sectors is encouraging. This approach allows for dietary interventions to be customized based on individual genetic, metabolic, and environmental profiles. The ISNN suggests that PN, which includes the consideration of genotype, can be more effective in improving public health and may be beneficial in long-term weight control [12]. The use of omics technologies could enable health professionals to provide tailored dietary recommendations and personalized advice, potentially revolutionizing public health guidelines through the incorporation of PN [12]. However, there are challenges to be addressed for the successful implementation of PN. This calls for continued progress in the development of tools for interpreting data, ensuring that the information derived from various tests and technologies is effectively communicated to consumers [12]. Additionally, there is an urgent need for the development of ethical and legal frameworks to facilitate the adoption and implementation of the newly introduced concepts of precision nutrition and medicine, ensuring their broad and individualized application [12]. The International Life Sciences Institute’s North American Branch has suggested a set of guiding principles for applying PN strategies. These guidelines highlight the importance of using individual-specific, evidence-based information to encourage changes in dietary behaviors, which could lead to measurable health improvements [80]. These guidelines aim to lay the groundwork for responsible, evidence-based research and practice in PN, and they encourage ongoing public discussion [80]. In conclusion, although PN presents potential benefits for public health, there is a need for additional research to enhance the accuracy of dietary intake assessments, improve and standardize systems approaches, and refine the application and dissemination of findings [80]. It is crucial to integrate the evolving area of PN with public health nutrition approaches to enhance diet quality and prevent chronic diseases.

## 9. Conclusions

The potential of PN to revolutionize dietary recommendations is profound and multifaceted. By integrating genetic, phenotypic, and environmental data, PN offers a nuanced approach to diet and health, promising improved outcomes by tailoring dietary advice to individual biological profiles. This individualized approach is especially pertinent in addressing chronic diseases such as obesity, cardiovascular disease, and diabetes, where one-size-fits-all dietary guidelines have shown limitations. As evidenced in the studies highlighted, PN not only enhances the precision of dietary interventions but also aligns with the increasing consumer demand for customized health solutions.

However, the realization of PN’s full potential necessitates sustained collaborative efforts across various domains. There is a pressing need for further research to refine our understanding of gene–diet interactions and to validate the efficacy of PN interventions in diverse populations. This research should be underpinned by advances in genomics, bioinformatics, and biotechnology which continue to evolve at a rapid pace. Collaboration among scientists to share data and insights, clinicians to apply these findings responsibly in patient care, and policymakers to create supportive regulatory frameworks is crucial. These combined efforts will ensure that PN not only progresses scientifically but also becomes ethically and practically implementable on a wide scale.

Reflecting on the balance between the benefits and risks of personalized dietary advice, it is clear that while the benefits hold significant promise, the risks cannot be overlooked (Table 5). The ethical, legal, and social implications, particularly concerning data privacy, informed consent, and the potential for exacerbating health disparities, must be rigorously managed. It is also imperative to critically evaluate the clinical relevance of genetic testing in nutrition and to ensure that such interventions do not oversimplify the complexities of human nutrition and health.

In conclusion, as we stand on the brink of a dietary revolution fueled by PN, it is essential to navigate this emerging field with a balanced perspective, acknowledging its transformative potential while conscientiously addressing the associated risks. The future of PN will significantly depend on our ability to integrate scientific innovation with ethical responsibility and inclusivity.

## Figures and Tables

**Table 1:** Comparison of traditional vs. personalized nutrition approaches.

Traditional Nutrition		Personalized Nutrition
West-established guidelines Broad applicability Cost-effective Simplicity	Advantages	Individualized approach Potential for improved health outcomes Enhanced adherence Integration of advanced technologies
Lack of individualization Generalized recommendations Outdated information Limited flexibility	Limitations	Higher costs Complexity Limited availability Evolving science Potential privacy concerns

**Table 2:** Overview of key genes involved in nutrigenomics.

Gene Name	Function	Associated Nutritional Influence	Relevant Studies and Findings
MTHFR	Methylenetetrahydrofolate reductase	Folate metabolism	Variations affect folate metabolism and cardiovascular risk [20,21,22]
APOE	Apolipoprotein E	Lipid metabolism	Influences lipid levels and cardiovascular disease risk [25,26,27]
TCF7L2	Transcription factor 7-like 2	Type 2 diabetes risk	Associated with increased risk of type 2 diabetes and response to dietary carbohydrates [29,30]
BCMO1	Beta-carotene oxygenase 1	Beta-carotene metabolism	Variations affect vitamin A levels and carotenoid metabolism [23,24]
FTO	Fat mass and obesity-associated protein	Obesity, energy balance	Linked to increased risk of obesity and response to dietary fats [31,32]

**Table 3:** Summary of personalized interventions.

Intervention Type	Target Population	Genetic Markers Used	Dietary Adjustments	Health Outcomes
Dietary modification	Obese individuals	FTO, MC4R	Reduced fat intake, increased physical activity	Weight loss, improved metabolic health
Supplementation	Individuals with folate deficiency	MTHFR	Increased folate intake	Reduced cardiovascular risk
Lifestyle changes	People at risk of cardiovascular disease	APOE	Modified fat intake, increased omega-3 fatty acids	Improved lipid profile
Medical nutrition therapy	Diabetics	TCF7L2, PPARG	Controlled carbohydrate intake	Better blood sugar control
Technological integration	General population	Various	Personalized meal plans based on genetic tests	Overall health improvement

**Table 4:** Technologies used in personalized nutrition.

Technology Type	Description	Application in Nutritional Assessment	Advantages and Limitations
Genomic Sequencing	Analyzing genetic variations	Identifying genetic predispositions	High accuracy, cost-intensive
Wearable Sensors	Monitoring physical activity and health metrics	Tracking lifestyle factors	Real-time data, privacy concerns
Bioinformatics Tools	Interpreting genetic data	Integrating multi-omics data	Comprehensive analysis, complex interpretation
AI and Machine Learning	Predicting health outcomes	Customizing nutrition plans	Predictive capabilities, ethical issues
Mobile Health Apps	Providing dietary recommendations	Engaging users in dietary changes	User-friendly, variable reliability

**Table 5:** Risk and benefits of Personalized Nutrition.

Potential Benefits	Associated Risks	Mitigation Strategies	Ethical Considerations
Optimized health outcomes	Data privacy concerns	Robust data security measures	Informed consent
Reduced risk of chronic diseases	Potential for misuse of genetic information	Strict regulatory frameworks	Transparency in data use
Improved dietary adherence	Ethical issues	Ethical guidelines	Fair access to PN services
Tailored interventions	Accessibility and cost	Insurance coverage, subsidies	Avoidance of genetic discrimination
Enhanced patient engagement	Over-reliance on genetic data	Comprehensive health assessment	Holistic approach to health

## Data Availability

No new data were created or analyzed in this study. Data sharing is not applicable to this article.
